# Supplementary material for: The influence of the brittle-ductile transition zone on aftershock and foreshock occurrence
Source: Nat Commun. 2020 Jun 15;11:3010. doi: 10.1038/s41467-020-16811-7 (PMC7295783; doi:10.1038/s41467-020-16811-7)
Supplement: Supplementary file 1 — Supplementary Information [file 41467_2020_16811_MOESM1_ESM.pdf]

Supplementary Note: The influence of the brittle-ductile transition zone on aftershock and foreshock occurrence

Petrillo G.[1], Lippiello E. [1], Landes F. [2] & Rosso A. [3]

[1]Department of Mathematics and Physics, University of Campania "L. Vanvitelli", 81100 Caserta, Italy.

[2]TAU, CNRS, INRIA, Univ. Paris-Sud, Université Paris-Saclay, 91405 Orsay, France

[3]LPTMS, CNRS, Univ. Paris-Sud, Université Paris-Saclay, 91405 Orsay, France

Corresponding author: E. Lippiello, `eugenio.lippiello@unicampania.it`

Supplementary Notes 1

In this supplementary materials we present data for different system size L and for different values of ϵ, keeping fixed the other parameters $\Theta = 0.5$ and $\sigma = 5$.

In Suppl. Fig.1 we plot the magnitude distribution for fixed Θ, σ and ϵ and for different value of the system size L. Results clearly show that, for the considered ϵ value, size effects are absent and, in particular, the upper magnitude cut-off m_U is not affected by L. We find (not shown) that also the other statistical features, investigated in the main text, do not depend on the system size L.

In Suppl. Fig.2 we plot the magnitude distribution for $\Theta = 0.5$, $\sigma = 5$, $L = 1000$ and for different values of ϵ. We observe that at small magnitudes $P(m)$ follows the GR-law with a b-value, $b \simeq 1.06$, which is quite independent on ϵ. The GR behavior extends up to the upper cut-off magnitude m_U which, conversely, clearly depends on ϵ. In order to investigate the dependence of m_U on ϵ, in Suppl. Fig.3 we plot $P(m) \times 10^{-bm}$, with $b = 1.06$, versus $m - m_U(\epsilon)$, where $m_U(\epsilon)$ is identified as the value which provides the best data collapse. Results of Suppl. Fig.3 show that curves for different ϵ collapse on the same master curve, supporting the previous observation that ϵ does not affect the b-value and only affects m_U. Deviation from the scaling collapse is only observed for data with the smallest considered $\epsilon = 0.001$. Data for $\epsilon = 0.001$, indeed, from one side present significant fluctuations around the initial constant behavior, from the other side present a peak at large magnitude. The presence of the peak can be a attributed to a finite size effect since this peak corresponds to earthquakes spanning over the whole system, $A \sim L^2$. The $m_U(\epsilon)$ providing the best data collapse appears to logarithmically depend on ϵ, $m_U(\epsilon) = -1.65 - 1.5 log(\epsilon)$, as shown by the best-fit in the inset of Suppl. Fig.3.

In Suppl. Fig.4a and in Suppl. Fig.4b we show that also the other statistical features of synthetic catalogs appear quite independent on ϵ, up to magnitude values $m < m_U$.

We finally remark that results of Suppl. Fig.s 2-4 suggest that, as in standard critical phenomena, the parameter ϵ quantifies the distance to the critical point introducing a characteristic length scale L_ϵ, which diverges when $\epsilon \to 0$. If one considers earthquakes of size smaller than L_ϵ, the system behaves as at the critical point whereas deviation from criticality are observed only at length scales larger than L_ϵ. This feature is also clearly observed in the qEW model (a class II model according to the definition of Section "Comparison with previous spring-block models") and well understood in the framework of the theory of the depinning transi-

tion. The situation is conversely different for the OFC model ($\Theta = 0$ and $\sigma = 0$) where the b-value of the GR law exhibits a clear dependence on ϵ.

Supplementary Note 2: Spatial organization of aftershocks

For each mainshock with magnitude $m_M > 3.25$ we evaluate the slip profile, i.e. the distribution of the number of slips n_j of each block during an earthquake. We then identify the peak slip n_{max}, defined as the maximum value of n_j, and define the mainshock slip contour as the border of the region where $n_j > \chi n_{max}$. Following [1] we set $\chi = 0.15$ and define Δr_i as the distance from the mainshock slip contour of the i-th aftershock hypocenter. Negative values of Δr_i indicate positions of aftershocks that locate within the slip contour, whereas positive values indicate aftershocks outside the slip contour. We also define the mainshock area A_χ as the area of the region enclosed within the mainshock contour with its radius given by $R_\chi = \sqrt{A_\chi/\pi}$.

The distribution of $\Delta r/R_\chi$ is plotted in Suppl. Fig.5 for a system with $L = 1000$, $\Theta = 0.5$, $\sigma = 5$ and $\epsilon = 0.008$, using data for 1000, $m > 3.25$ mainshocks. We find a behavior very similar to the one obtained by [1] for 101 large, magnitude $m > 7$, instrumental mainshocks. Indeed, as in ref. [1], we find that the majority of aftershock hypocenters are located near or beyond the outer edge of the mainshock contour. Only about the 1% of the aftershock are located in the internal region $\Delta r < 0.7R_\chi$, whereas about the 25% of aftershocks are located outside the mainshock contour at a distance $\Delta r > 1.2R_\chi$. The number of external aftershocks is a decreasing function of the distance from the mainschock contour and only the 3% of aftershocks are found at distance larger than two source dimensions.

Supplementary Figures

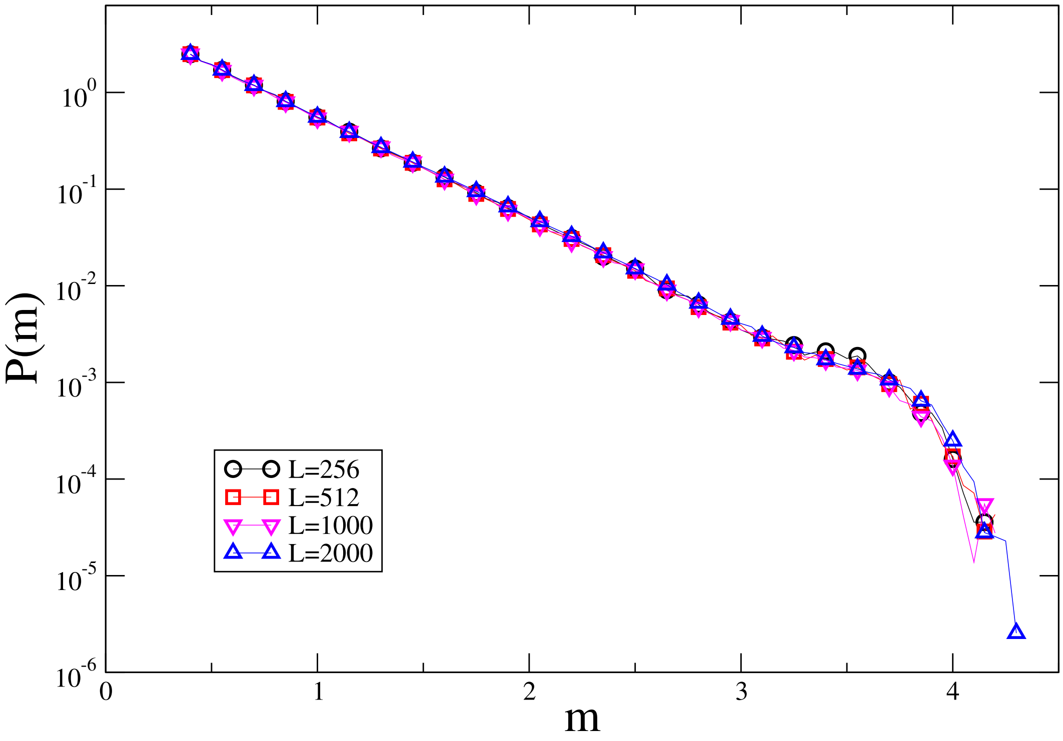

Figure 1: **The magnitude distribution as function of the system size L.** The magnitude distribution for $\Theta = 0.5$, $\sigma = 5$, $\epsilon = 0.008$ and different values of the system size L.

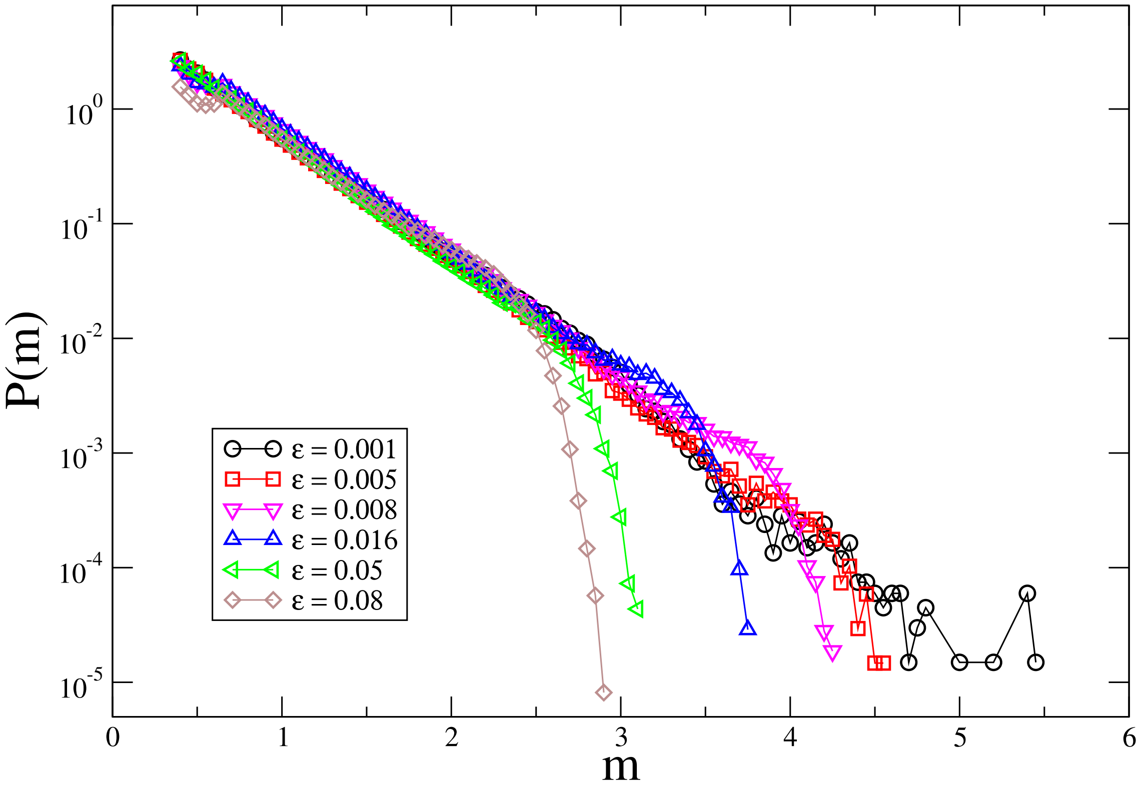

Figure 2: **The magnitude distribution as function ϵ.** The magnitude distribution for $\Theta = 0.5$, $\sigma = 5$, $L = 1000$ and different values of ϵ.

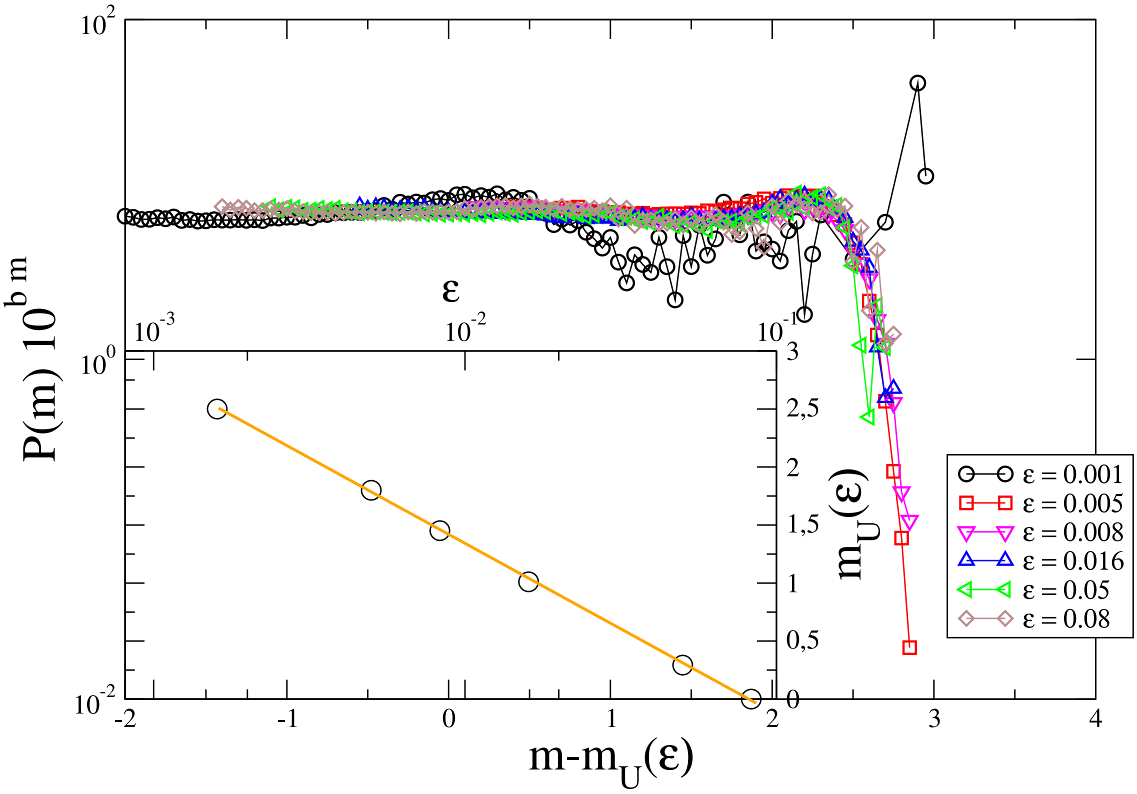

Figure 3: **The magnitude distribution as function of $m - m_U(\epsilon)$.** The magnitude distribution for $\Theta = 0.5$, $\sigma = 5$, $L = 1000$ is multiplied by $10^{1.06m}$ and plotted versus $m - m_U(\epsilon)$. The value of $m_U(\epsilon)$ is plotted in the distance as function of ϵ. The orange line is the best fit $m_U(\epsilon) = -1.65 - 1.5log(\epsilon)$.

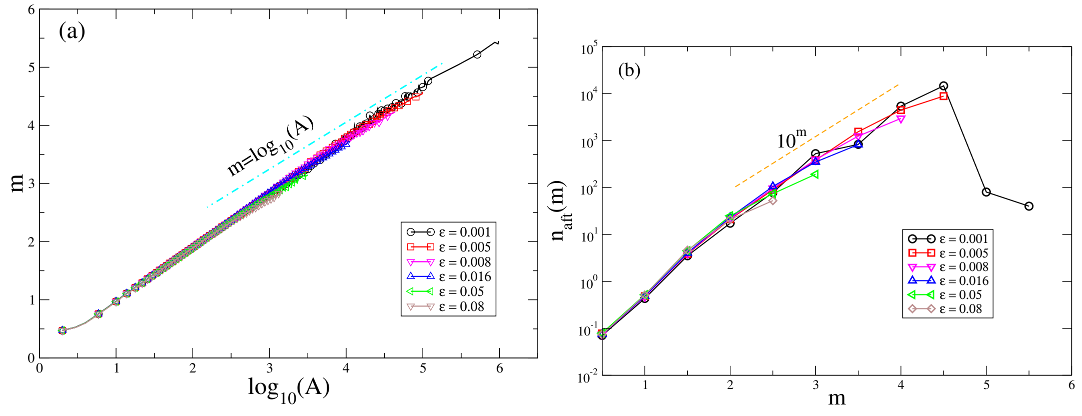

Figure 4: **Statistical features as function ϵ.** The $m - logA$ scaling (panel a) and the number of aftershock versus the magnitude (panel b) for a system with $\Theta = 0.5$, $\sigma = 5$, $L = 1000$ and different values of ϵ. The cyan dot-dashed line (panel a) is the linear fit $m = \gamma_0 log_{10} A$, with $\gamma_0 = 1$, and the orange dashed line (panel b) is the productivity law $n_{aft}(m) \sim 10^{\alpha m}$, with $\alpha = 1$.

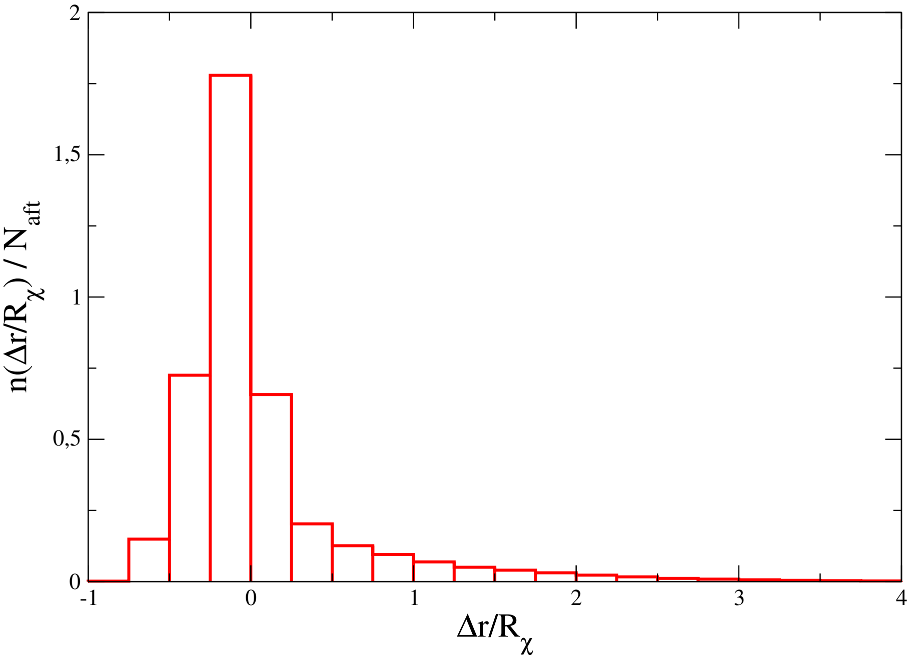

Figure 5: **Distribution of hypocentral distances from the mainshock contour.** The histogram of aftershock occurring at a distance $\Delta r/R_\chi$ from the mainshock, divided by the total aftershock number N_{aft}. We consider aftershock sequences after 1000, $m_M > 3.25$ mainshocks, for a synthetic catalog with $\Theta = 0.5$, $\sigma = 5$, $L = 1000$ and $\epsilon = 0.008$.

Supplementary References

[1] Wetzler, N., T. Lay, E. E. Brodsky, and H. Kanamori (2018), Systematic deficiency of aftershocks in areas of high coseismic slip for large subduction zone earthquakes, *Science Advances*, *4*(2),
